# Possible association of diazotrophs with marine zooplankton in the Pacific Ocean

**DOI:** 10.1002/mbo3.385

**Published:** 2016-06-28

**Authors:** Kazi Md. Azimuddin, Junya Hirai, Shotaro Suzuki, Md. Nurul Haider, Aiko Tachibana, Keigo Watanabe, Minoru Kitamura, Fuminori Hashihama, Kazutaka Takahashi, Koji Hamasaki

**Affiliations:** ^1^ Atmosphere and Ocean Research InstituteThe University of TokyoKashiwaChibaJapan; ^2^ National Research Institute of Fisheries ScienceFisheries Research AgencyYokohamaKanagawaJapan; ^3^ Japan Agency for Marine‐Earth Science and TechnologyYokosukaKanagawaJapan; ^4^ Department of Ocean SciencesTokyo University of Marine Science and TechnologyMinato‐KuTokyoJapan; ^5^ Department of Aquatic ScienceGraduate School of Agriculture and Life SciencesThe University of TokyoBunkyo‐KuTokyoJapan

**Keywords:** Marine, microorganisms, *nifH*, Pacific Ocean, zooplankton

## Abstract

Dinitrogen fixation, the biological reduction in N_2_ gas to ammonia contributes to the supply of new nitrogen in the surface ocean. To understand the diversity and abundance of potentially diazotrophic (N_2_ fixing) microorganisms associated with marine zooplankton, especially copepods, the *nifH* gene was studied using zooplankton samples collected in the Pacific Ocean. In total, 257 *nifH* sequences were recovered from 23 *nifH*‐positive DNA extracts out of 90 copepod samples. The *nifH* genes derived from cyanobacteria related to *Trichodesmium*,* α*‐ and *γ*‐subdivisions of proteobacteria, and anaerobic euryarchaeota related to *Methanosaeta concilii* were detected. Our results indicated that *Pleuromamma*,* Pontella,* and *Euchaeta* were the major copepod genera hosting dinitrogen fixers, though we found no species‐specific association between copepods and dinitrogen fixers. Also, the digital PCR provided novel data on the number of copies of the *nifH* gene in individual copepods, which we report the range from 30 to 1666 copies per copepod. This study is the first systematic study of zooplankton‐associated diazotrophs, covering a large area of the open ocean, which provide a clue to further study of a possible new hotspot of N_2_ fixation.

## Introduction

Primary production in the marine environment is thought to be nitrogen (N) limited, as bioavailable N is often present at concentrations below that of other necessary elements. Although many organisms can assimilate N in combined forms like ammonium (${\text{NH}}_{4}^{+}$), nitrate (${\text{NO}}_{3}^{-}$), nitrite (${\text{NO}}_{2}^{-}$), or urea, the most abundant form, dinitrogen gas (N_2_), is generally inaccessible to all but some prokaryotes carrying the enzyme complex nitrogenase, capable of reducing N_2_ gas to ammonium (${\text{NH}}_{4}^{+}$) (Bullen and LeComte 1972; Zehr et al. 2008). The activity of this enzyme is inhibited by oxygen (Gallon 1981).

N_2_ fixation is a key biological process that provides new N to the marine ecosystems (Zehr and Ward 2002). An imbalance in the N budget is currently debated. Galloway et al. (2004) did not calculate an N imbalance. However, other estimates of the global ocean N budget suggest the inputs and outputs of N_2_ gas are unbalanced, that a deficit of N_2_ fixation exists in which N losses far‐exceed gains (Codispoti 2007), and that N_2_ fixation rates are likely underestimated (Mohr et al. 2010). Therefore, it is essential to assess the presence, diversity, distribution, and abundance of N_2_–fixers, which may help to arrive at better estimates of N_2_ fixation.

Molecular analyses of *nifH* genes, those encoding enzymes involved in the fixation of atmospheric nitrogen into a form of nitrogen available to living organisms have been performed in studies examining the presence and diversity of N_2_–fixers in oceanic environments (Zehr et al. 1998). The most significant N_2_ fixing microorganisms were thought to be filamentous cyanobacteria, such as *Trichodesmium* (Capone et al. 1997), symbiotic and free‐living unicellular cyanobacteria (UCYN, including *Crocosphaera*) (Zehr et al. 2001), and filamentous cyanobacteria of the order *Nostocales* that associate with diatoms (Carpenter et al. 1999). However, diverse *nifH* genes clustering into noncyanobacterial clades have also been reported from the surface waters down to below the euphotic zone in the open ocean (Zehr et al. 2003; Langlois et al. 2005; Riemann et al. 2010; Farnelid et al. 2011; Moisander et al. 2014). Although diazotrophic bacteria obtained from the open ocean have clustered into a wide range of bacterial groups, including *α*‐, *β*‐, *γ*‐, and *δ*‐ proteobacteria and *Firmicutes* (Zehr et al. 2003), it has been speculated that many presently unidentified organisms may be active in ocean biological N_2_ fixation.

As *nifH* genes have been previously reported from copepods, an association between them and N_2_ fixing bacteria has already been demonstrated (Zehr et al. 1998; Braun et al. 1999; Scavotto et al. 2015). Our hypothesis built on this proposed association is that the guts of copepods provide a perfect anaerobic environment where N_2_‐fixers could flourish. Whereas N_2_ fixation by microbes in the alimentary (gut) tract of many terrestrial arthropods (insects) serves as a source of N (Ohkuma et al. 1996), few species of N_2_ fixing organism have been identified or isolated from marine planktonic‐arthropods (Proctor 1997; Zehr et al. 1998; Braun et al. 1999; Scavotto et al. 2015). Copepods, small marine arthropod crustaceans ranging in size from species <1 mm in length to those more than 10 mm, dominate mesozooplankton within the upper 600 m of the world oceans (Star and Mullin 1981), wherein they occur at densities typically ranging 1–100 individuals m^−3^ (Longhurst 1985). N_2_ fixation in zooplankton‐associated microbial communities could provide an important, little understood source of possible new N in the open ocean. Herein, we report novel data searching the presence, diversity, distribution, and abundance of possible N_2_‐fixers associated with marine zooplankton over a wide area of the Pacific Ocean.

## Materials and Methods

### Study area and sample collection

**Figure 1:**
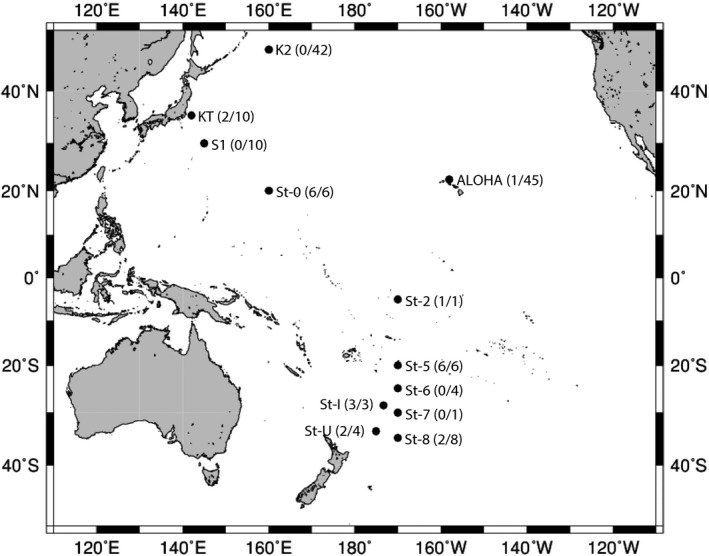
Zooplankton sampling locations 2011–2014: values (in parentheses) indicate the number of *nifH*‐positive zooplankton samples over the total number of samples analyzed at each site.

In total, 140 zooplankton samples were collected from 12 locations in the Pacific Ocean during the cruises of R/V *Mirai* (MR‐11‐2), R/V *Hakuho‐maru* (KH‐11‐10 and KH‐13‐7), and R/V *Shinsei‐maru* (KS‐13‐T2) between December 2011 and January 2014 (Table 1; Fig. 1). The K2 and S1 stations were located in the subarctic and subtropical North Pacific, respectively. The ALOHA station was located in the subtropical North Pacific. The KT station was located in the Kuroshio Current area. The other eight stations were located in the tropical and subtropical areas of the South Pacific. Samples were collected by North Pacific Standard Net (NORPAC net, 100 *μ*m mesh) towed at 0.7 m s^−1^, vertically hauled from a depth of 200 m to the surface (200–0 m). Net contents were washed with 0.2 *μ*m‐filtered seawater. Selected copepod samples from station 5, hereafter referred to as “empty‐gut” copepods, were incubated for 72 h in 0.2 *μ*m filtered seawater to evacuate gut contents. From sample KT‐8 only gut of the respective copepod was separated aseptically referred as only “gut sample.” Zooplankton was sorted and identified using conventional light microscopy (Leica Wild MZ‐8, Vashaw Scientific, Inc., Norcross, GA) and taxonomic guides, then stored individually at −30°C until analysis.

**Table 1:** Zooplankton sampling site environmental parameters (0–200 m). Salinity, temperature and dissolved oxygen (DO) (0–200 m) presented as ranges; surface macronutrient (N+N = ${\text{NO}}_{3}^{-}\;+\;{\text{NO}}_{2}^{-}$; ${\text{PO}}_{4}^{3-}$) concentrations at 10 m

Cruise	Station	Latitude, longitude	Date	Salinity (psu)	Temp (°C)	DO (mL L^−1^)	N+N (n mol L^−1^)	${\text{PO}}_{4}^{3-}$ (nmol L^−1^)
MR‐11‐2	S1	30°N, 145°E	18/02/2011	34.69–34.72	17.77–18.31	0.18–0.23	500	ND
	K2	47°N, 160°E	02/03/2011	32.91–33.81	01.80–03.40	0.19–0.33	21550	ND
KH‐11‐10	ALOHA	22°46′N, 158°05′W	19/11/2011	34.81–35.34	17.76–24.26	4.25–4.55	90	40
KS‐13‐T2	KT	35°50′N, 142°20′E	14/10/2013	34.18–34.70	13.61–26.13	3.79–4.54	ND	ND
KH‐13‐7	St‐0	20°N, 160°E	16/12/2013	34.82–35.16	18.39–27.72	2.94–4.24	6	12
St‐2	5°05′S, 170°W	25/12/2013	35.67–35.68	20.47–29.12	2.86–4.27	75	545
St‐5	20°S, 170°W	03/01/2014	35.45–35.62	21.81–27.73	4.07–4.69	4	129
St‐6	25°S, 170°W	07/01/2014	35.53–35.55	18.89–26.23	4.02–4.88	ND	30
St‐7	30°S, 170°W	08/01/2014	35.29–35.43	14.89–24.00	4.37–5.35	4	30
St‐8	35°S, 170°W	11/01/2014	35.21–35.23	14.32–21.67	4.42–5.45	3	77
St‐I	28°47′S, 173°30′W	13/01/2014	23.87–25.90	16.86–24.41	4.19–5.09	ND	ND
St‐U	33°07′S, 175°05′W	18/01/2014	24.29–26.32	14.29–23.16	4.31–5.48	ND	ND

ND, no data.

### Hydrographic parameters and nutrients

Samples for salinity, dissolved oxygen (DO), and macronutrient analysis (${\text{NO}}_{3}^{-},$ ${\text{NO}}_{2}^{-}$ and ${\text{PO}}_{4}^{3-}$) were collected in acid‐cleaned Teflon‐coated 12‐L Niskin‐X bottles on a CTD (Conductivity, Temperature, Depth) carousel system attached at the end of titanium‐armored cable. Temperature and salinity profiles were determined with SBE 911 (Sea‐bird Electronics, Inc., Bellevue, WA). DO concentrations were measured by Winkler titration using an automatic titrator (806 Titrando, Metrohm AG, Herisau, Switzerland). During the R/V *Hakuho‐maru* cruises, nanomolar macronutrient concentration levels were determined by liquid wave guide spectrophotometer (Hashihama et al. 2009), for which detection limits of ${\text{NO}}_{3}^{-}\;+\;{\text{NO}}_{2}^{-}$ and ${\text{PO}}_{4}^{3-}$ were 3 nmol L^−1^, whereas those made during the R/V *Mirai* cruise were determined by standard colorimetric methods.

### Molecular analysis of *nifH*

DNA was directly extracted from a copepod and its associated microorganisms using a Qiagen Blood and Tissue Kit (Qiagen, Hilden, Germany), in accordance with manufacturer protocols. Six samples from KH‐27 to KH‐32 represent extracts from copepods with an empty‐gut and the KT‐8 represents an extract only from the gut of the respective copepod (Table 2). The DNA concentration of samples was measured using a Quant‐iT^™^Picogreen^®^ dsDNA Reagent and Kit (Invitrogen, Carlsbad, CA) and Microplate reader (SH‐9000; Corona Electric, Ibraraki, Japan). Partial *nifH* fragments were amplified from the DNA of an individual sample by the nested PCR (Zehr et al. 2001). Two degenerate oligonucleotide PCR primer sets were used to amplify approximately 350‐bp segments of the *nifH* gene (Zehr and McReynolds 1989; Zani et al. 2000). Each reaction contained 0.05 *μ*L of 5U *μ*L^−1^ EX Taq HS (TaKaRa, Tokyo, Japan), 1.0 *μ*L of 10× ExTaq Buffer (TaKaRa), 0.8 *μ*L of 0.2 mmol L^−1^ dNTP mixture (TaKaRa), 1.0 *μ*mol L^−1^ each primer, 2 *μ*g BSA (TaKaRa), and 1 *μ*L of template DNA in a final volume of 10 *μ*L. The first and second PCRs were run in triplicate; the first, 40 cycles at 95°C for 3 min, followed by the second, 35 cycles at 98°C for 10 sec, 54°C for 30 sec, 72°C for 30 sec, with a final extension at 72°C for 7 min. Amplification of *nifH* was checked using 3 *μ*L of the second PCR product using 1.5% agarose gel. No visible band was detected from the negative control in PCR reactions. Samples that produced a 350‐bp visible product were extracted and gel purified (Qiagen PCR purification kit) and cloned using TOPO TA Cloning Kit (Invitrogen) for sequencing, transferred into *E. coli* DH5*α* competent cells (TaKaRa).

**Table 2:**
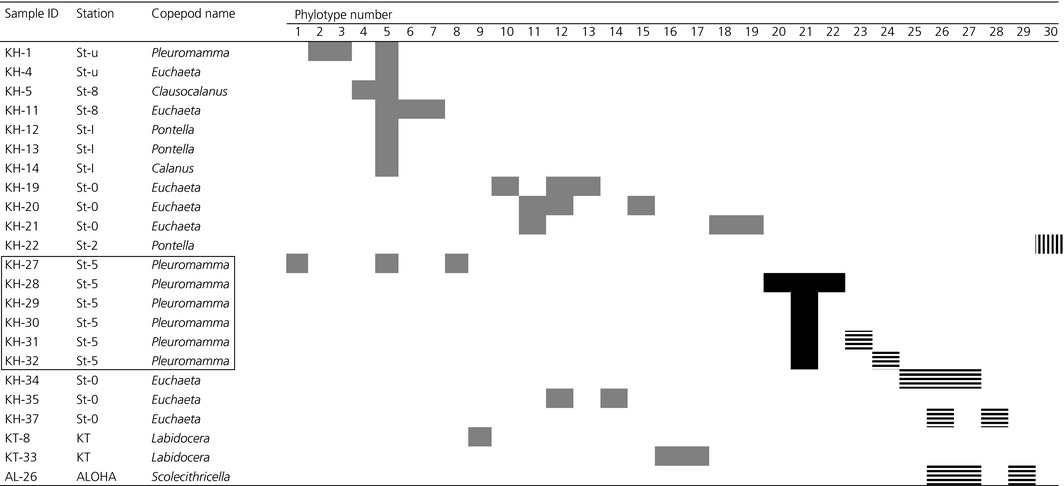
*nifH*‐positive copepod genera from which 257 *nifH* clones are attributed to 30 phylotypes: light shaded box (*γ*‐proteobacterial), dark solid box (*α*‐proteobacterial), vertical line in the box (archeal), and horizontal line in the box (cyanobacterial; see Fig. 2). Samples inside the square box indicate the copepods with an empty‐gut

Clones were screened to identify those with the correct insert, and 24 colonies from each sample were selected for DNA sequencing with the BigDye v.3.1 Sequencing Kit (Applied Biosystems, Foster City, CA). Sequencing of *nifH* was performed using an ABI 3130 genetic analyzer in accordance with manufacturer protocols. Vector and primer sequences were removed manually. The *nifH* sequences were analyzed by BLAST searches against the National Center for Biotechnology Information (NCBI) database. Clones sharing 100% similarity in amino acid sequences were clustered into the same phylotype using the CD‐HIT program (Huang et al. 2010). Two hundred and fifty‐seven representative sequences were aligned with MUSCLE (Edgar 2004) in the Molecular Evolutionary Genetics Analysis (MEGA ver. 5) software package (Tamura et al. 2011). The closest sequences of uncultured and cultured bacteria identified from BLAST comparisons were added to the dataset. A distance‐based neighbor‐joining phylogenetic tree was constructed. Bootstrap values were determined from 1000 iterations.

### Quantification of *nifH*

**Figure 2:**
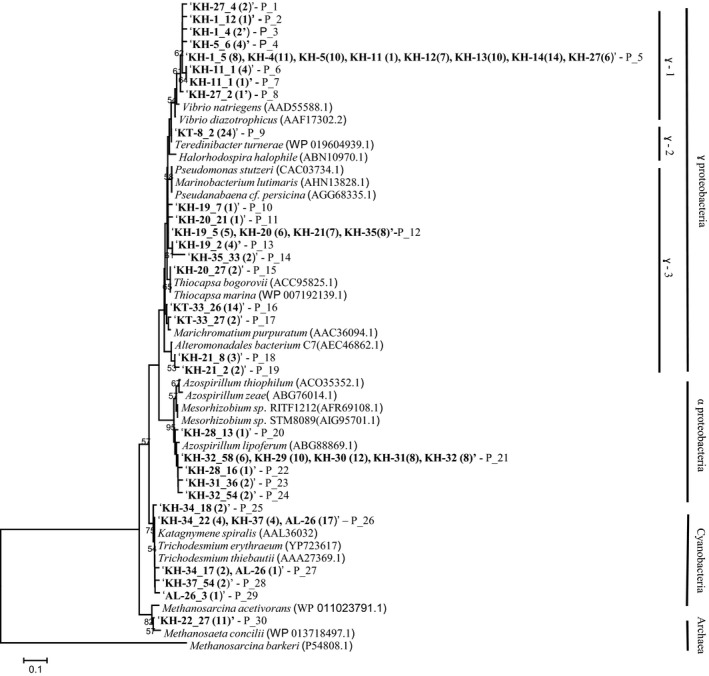
Phylogenetic tree constructed based on analysis of 257 *nifH*‐translated amino acid sequences using neighbor‐joining method. Bold type denotes sequences in this study (sequences with 100% similarity are grouped); boldface numbers (in parentheses) represent the number of retrieved clones from each copepod. Bootstrap values (>50%) are indicated at branch points; scale bars are estimated sequence divergences (10%); and P, phylotype.

For determining the copy number of *nifH* genes associating with an individual copepod, we used droplet digital PCR (ddPCR) system (Bio‐Rad Laboratories, Hercules, CA). Six specific primer and probe sets were designed to detect the *nifH* gene from different clusters (Fig. 2; Table 3) using GenScript Real‐time PCR primer design software (https://www.genscript.com/ssl-bin/app/primer). Among *γ* proteobacterial *nifH* sequences, three subclades were designated (*γ* − 1, *γ* − 2 and *γ* − 3). From these three subclades, three reference sequences were selected for the design of primers and probes. From *α*‐proteobacteria, cyanobacteria and archaea, one reference sequence from each clade was selected (Table 3). Specificity of these six primer and probe sets was tested by applying them to our cloned *nifH* sequences in qPCR (DNA Engine OPTICON2, Bio‐Rad Laboratories). Standards for the different *nifH* clusters were achieved by cloning *nifH* amplicons using the TOPO TA Cloning Kit (Thermo Fisher Scientific Inc, Waltham, MA). Plasmid DNA was purified using PureLink Quick Plasmid Miniprep Kit (Thermo Fisher Scientific Inc, Waltham, MA) in accordance with manufacturer protocols. Plasmid concentration were determined by Quant‐iT^™^ Picogreen dsDNA Reagent and Kit (Thermo Fisher Scientific Inc) and a Microplate reader (SH‐9000; Corona Electric). The number of *nifH* genes (copies *μ*L^−1^) was determined using ddPCR system, in accordance with manufacturer protocols. Each PCR reaction contained 10 *μ*L 2× ddPCR supermix, 1.8 *μ*L of 10 *μ*mol L^−1^ forward and reverse primer, 0.5 *μ*L of 10 *μ*mol L^−1^ probe, and 3 *μ*L of template DNA in a final volume of 20 *μ*L. Droplets then were prepared by QX100 Droplet Generator (Bio‐Rad Laboratories). Duplicate runs were performed for all ddPCR. No template control was run in duplicate for each reaction. Cycling conditions of ddPCR were: 95°C for 10 m followed by 40 cycles of 94°C for 30 sec, 56°C for 60 sec and finally 98°C for 10 m. After PCR reaction, fluorescence measurement was performed by QX100 Droplet Reader (Bio‐Rad Laboratories). The copy number of *nifH* genes (copies *μ*L^−1^) was determined by QuantaSoft software (Bio‐Rad Laboratories). The digital PCR detects one copy in one reaction. We added 3 *μ*L of DNA extracts in one reaction solution. This is why the minimum copy number per a unit volume (*μ*L) was less than one. Because the final volume of DNA extracts obtained from an individual copepod was 50 *μ*L, the minimum copy number we could detect in this study was 17 copies in one copepod. The copy number per a unit volume (copies *μ*L^−1^) was multiplied by 50 for determining the total *nifH* copy number of the individual copepod.

**Table 3:** Primer and probe sets of ddPCR of *nifH* gene of *γ*‐proteobacteria, *α*‐proteobacteria, cyanobacteria, and archaeal clusters as described in Fig. 2

Type	Reference sequence	Forward primer 5′–3′	Probe 5′–3′	Reverse primer 5′–3′	Clone Accession number
ɣ‐proteobacteria (ɣ‐1)	KH‐1—5	ATTACACCGCGACCGGCACA	ATTACACCGCGACCGGCACA	GCCGAGTACGTCGTAGAACA	LC012983
ɣ‐proteobacteria (ɣ‐2)	KT‐8—2	ACCGATCCAGCTTCAGCAGCC	ACCGATCCAGCTTCAGCAGCC	CAGACCACCATCATGCATCT	LC013179
ɣ‐proteobacteria (ɣ‐3)	KH‐19‐5	TGCCTGCCTGAGCAGCCATT	TGCCTGCCTGAGCAGCCATT	TCGTCTTATCCTGCATTCCA	LC013057
*α*‐proteobacteria	KH‐32‐58	CGCCGGATTCCACGCATTT	CGCCGGATTCCACGCATTT	CAGGAAGTTGATCGAGGTGA	LC013151
Cyanobacteria	KH‐34‐22	TCTACATCTTCAACTGCACCGCGTT	TCTACATCTTCAACTGCACCGCGTT	TGTACTTCACGTTGCTGCTG	LC013156
Archaea	KH‐22‐27	TCAAATGCGTCGAGTCCGGC	TCAAATGCGTCGAGTCCGGC	AGTCCGATGGAGGTGATGAT	LC013091

### Nucleotide sequence accession numbers

Sequences recovered from this study have been deposited in the DNA Data Bank of Japan (DDBJ) with accession numbers LC012980–LC013236.

## Results

### Environmental parameters and nutrients

Site‐specific values for salinity (ranging 23.87–35.68), temperature (1.8–29.1°C), dissolved oxygen (0.18–5.48 mL L^−1^), N+N (${\text{NO}}_{2}^{-}\;+\;{\text{NO}}_{3}^{-}$) from 3 nmol L^−1^ to 2.1 *μ*mol L^−1^ and ${\text{PO}}_{4}^{3-}$ 12–545 nmol L^−1^, are presented in Table 1.

### Zooplankton taxonomy

The taxonomic composition of 140 zooplankton samples comprised 90 copepods, nine amphipods and polychaetes, eight arrow worms and krill, five shrimp larva, four doliolids, three mollusks, and two pteropods and salps (Tables S1 and S2). Among the 90 copepods, *nifH* genes were amplified from the DNA of 23 samples (Table 2; Fig. 1). The copepods hosting *nifH*‐positive microorganisms were identified as seven genera: *Euchaeta*,* Pleuromamma, Pontella*,* Labidocera*,* Clausocalanus*,* Calanus,* and *Scolecithricella* (Table 2).

### *nifH* detection and sequences

Of 52 zooplankton samples from the stations K2 and S1, including 24 copepods, none was *nifH* positive (Fig. 1). Of 45 samples from the station ALOHA, including 23 copepods, only one copepod was *nifH* positive. Accordingly, copepods alone were selected for detecting *nifH* genes in subsequent cruises (KH‐11‐10, KS‐13‐T2 and KH‐13‐7). Of 10 copepods from the station KT, two proved *nifH* positive, whereas 20 out of 30 copepods from the other stations (0, 2, 5–8, U, I) were *nifH* positive (Fig. 1).

Totally 257 *nifH* clones obtained from 23 *nifH*‐positive samples were sequenced. The sequences were clustered into 30 phylotypes (Fig. 2). Most (163 out of 257) *nifH* sequences were grouped with *γ*‐proteobacterial ones. Many of them (82) were from the subtropical South Pacific samples (KH: 1, 4, 5, 11–14 and 27) and were closely related to *nifH* sequences of *Vibrio natriegens* and *V. diazotrophicus* (Fig. 2). Also, 24 *γ*‐proteobacterial sequences from the Kuroshio region (sample KT‐8) were related to the *nifH* of *Teredinibacter turnerae*, whereas 36 *γ*‐proteobacterial sequences from the tropical North Pacific (samples from KH‐19 to KH‐21 and KH‐35) were related to the *nifH* sequences of *Thiocapsa bogorovii* and *T. marina* (Tourova et al. 2009). All 50 *α*‐proteobacterial sequences were recovered from samples of empty‐gut copepods from the tropical South Pacific (samples from KH‐28 to KH‐32) were closely related to the *nifH* of *Azospirillum lipoferum* (Doroshenko et al. 2007). Thirty‐three *nifH* sequences obtained from three copepods (AL‐26, KH‐34 and KH‐37) were grouped into a cluster of cyanobacteria (Zehr et al. 2003); 27 were related to the *nifH* of *Katagnymene spiralis* (Lundgren et al. 2001) and six were related to that of *Trichodesmium* (Zehr et al. 1998). Eleven sequences from the sample KH‐22 were closely related to the *nifH* sequence of euryarchaeota, *Methanosaeta concilii* (Chien and Zinder 1996). No *nifH* sequences of unicellular cyanobacteria (UCYN‐A, UCYN‐B, and UCYN‐C) were identified from any sample.

### *nifH* abundance

**Figure 3:**
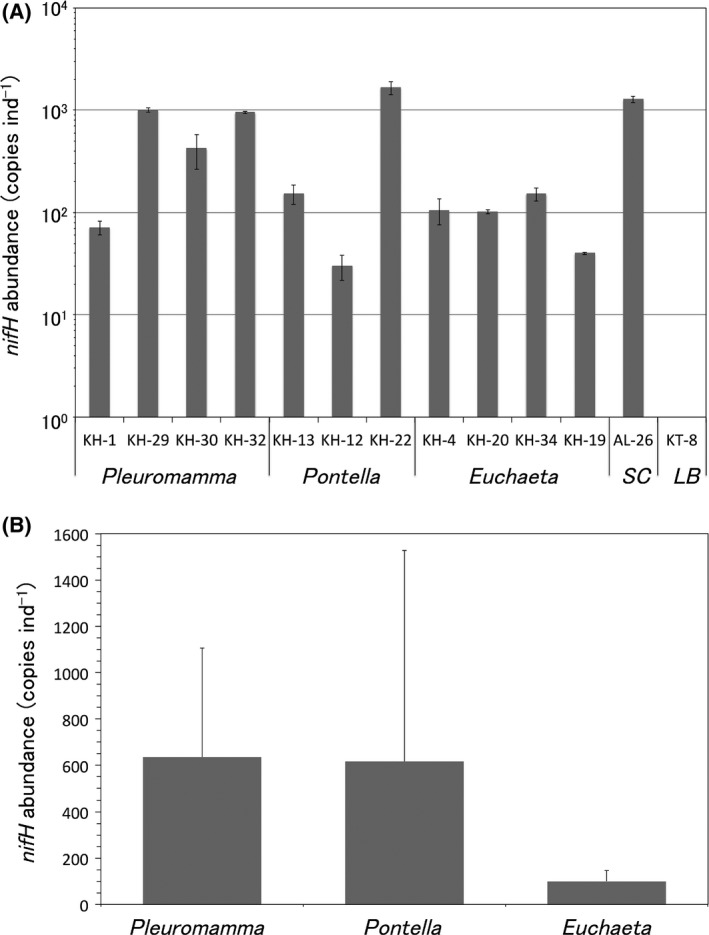
(A) Comparison of the *nifH* abundance of individual copepods (no *nifH* gene was detected from sample KT‐8). (B) Comparison of the average *nifH* copies of three copepod genera. SC,* Scolecithricella*; LB,* Labidocera*. Error bars indicate standard deviation.

**Figure 4:**
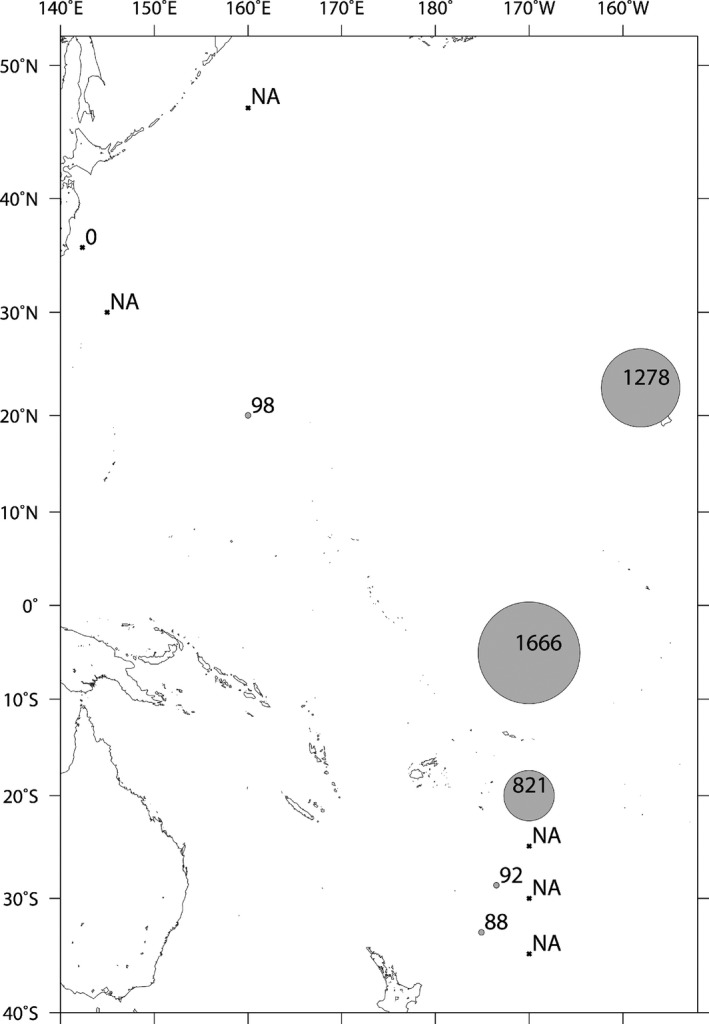
Average *nifH* copy number from individual copepods. Numerical values except 1666 and 1278 represent average *nifH* copy numbers determined by ddPCR. 1666 and 1278 are from single measurement each.

Of 23 *nifH*‐positive copepod samples, 13 (KH: 1, 4, 12, 13, 19, 20, 22, 29, 30, 32, 34; and KT‐8 and AL‐26) were selected for ddPCR analysis (Fig. 3A and B). Samples KH: 4, 19, 20, 34 contained *Euchaeta*; KH: 12, 13, 22, *Pontella*; KH: 1, 29, 30, 32, *Pleuromamma*; KT‐8, *Labidocera*; and AL‐26, *Scolecithricella* (Fig. 3A). *nifH* abundance could be determined from all but one (that of sample KT‐8 was possibly below the detection limit). Among these 12 samples the range of *nifH* copy number per copepod ranged 30–1666. Both the lowest and highest *nifH* copy numbers (30 and 1666 copepod^−1^) were recorded from samples of *Pontella* (Fig. 3A). The lowest number was recorded from the sample (KH‐12) of the subtropical South Pacific (St‐2). The highest number was recorded from the sample (KH‐22) of the tropical equatorial Pacific (St‐2). The *nifH* gene copy of cyanobacteria *Trichodesmium* was 1278 per copepod. (Fig. 3A and 4). The average *nifH* copy numbers in genera *Euchaeta*,* Pontella,* and *Pleuromamma* were 99.9 ± 45.9, 616.6 ± 911.3, and 634.1 ± 443, respectively (Fig. 3B). When the *nifH* copy number of an individual copepod was averaged at each stations, the numbers ranged from 88 to 1666 (Fig. 4).

## Discussion

Most of our sequences (213 of 257) were similar to those of heterotrophic bacteria, suggesting their dominance in copepod‐associating N_2_ fixing communities in oligotrophic tropical and subtropical Pacific Ocean waters (Fig. 2). *β*‐ and *γ*‐proteobacterial *nifH* sequences have been reported from calanoid copepods in the Gulf of Mexico (Zehr et al. 1998), *γ*‐proteobacterial *nifH* have been reported from copepods in coastal waters of the North Atlantic Ocean (Scavotto et al. 2015) and *α*‐, *β*‐ and *γ*‐proteobacterial *nifH* from copepods and euphausids in the Caribbean and western Bahama Sea (Braun et al. 1999). In this study, *γ*‐proteobacterial phylotypes were more abundant than *α*‐proteobacterial phylotypes.

Some of our cyanobacterial *nifH* sequences clustered to those of *Trichodesmium* and *K. spiralis*, which have been previously recovered from the seawater samples of the Pacific (Lundgren et al. 2001 and Orcutt et al. 2002), in addition to copepods from a shallow eutrophic lake (Yan et al. 2011). All clones recovered from the ALOHA station sample were clustered to reported cyanobacterial *nifH* sequences, suggesting the type of N_2_‐fixers associating with copepods are influenced by those in the surrounding water column.

Eleven *nifH* sequences recovered from *Pontella* in the equatorial tropical Pacific Ocean were clustered with the *nifH* sequence of *Methanosaeta concilii*, an obligate anaerobic methanogenic euryarchaeota (Patel and Sprott 1990). These were aligned in the cluster II *nifH* sequences described by (Zehr et al. 2003). *S*equences of this cluster have been recovered from anoxic environments including marine sediments, soils, and termite guts (Ueda et al. 1995; Zehr et al. 1995 and Ohkuma et al. 1996). It has been well‐known that methanogens are present in guts of copepods (Marty 1993; DeAngelis and Lee 1994). Also, some methanogens have reportedly possessed *nifH* genes and shown diazotrophic growth (Reeve 1992). Given these previously reported evidences, it is reasonably speculated that N_2_‐fixing methanogens reside within the copepod gut. Our finding of *nifH* sequences related to methanogens supports this speculation.

All *nifH* sequences from empty‐gut copepods at St‐5 except KH‐27 clustered with *Azospirillum* spp (Doroshenko et al. 2007) and related *α*‐proteobacteria (Fig. 2). Scavotto et al. (2015) found gamma proteobacterial *nifH* sequences and N_2_ fixing activity in empty‐gut copepods from coastal waters of the North Atlantic Ocean, suggesting these bacteria represent permanent diazotroph associations. Alpha proteobacteria found from empty‐gut copepods may be more permanently associating diazotrophs with copepods than other bacteria.

Abundance of diazotrophs associated with marine copepods may be related to host body size, as more were found in the larger *Pontella* than *Euchaeta*, of body size 5.9–6.2 mm, and 2.5–3.5 mm, respectively (Bradford‐Grieve 1999). Our results also suggest that the abundance of diazotrophs might vary within the copepod genus, given the highest and lowest number of copies of *nifH* genes were recovered from *Pontella* (Fig. 3A). A possible explanation is that the variation in *nifH* gene copy is related to the abundance of diazotrophs in waters in which copepods occur. The degree of diazotroph association may change depending on spatial variability in abundances of both copepods and diazotrophs in water.

Although marine diazotrophic abundance in water samples in the Pacific have been reported, ours is the first to quantify *nifH* gene copy number in individual copepods. Real‐time PCR was reportedly used to quantify UCYN‐A *nifH* copy number in copepod samples (Scavotto et al. 2015). Although 10 copepods (*Acartia*) were pooled to extract DNA or RNA, most of them were below detection and only one was detectable but not quantifiable. Generally speaking, ddPCR system provides orders of magnitude more precision and sensitivity than real‐time PCR (Hindson 2011). We did not try to use a conventional real‐time PCR in this study, however, ddPCR may be a better choice than the real‐time PCR to quantify low abundance of *nifH* gene in copepod samples. Abundance of *γ* proteobacterial *nifH* gene was reportedly 2.5 × 10^3^ in South Pacific waters (Mahaffey et al. 2005) and 6.8 × 10^3^ copies L^−1^ in North Pacific waters (Bombar et al. 2013). Abundance of *Trichodesmium nifH* gene was 1.4 × 10^5^ copies L^−1^ in South Pacific waters (Mahaffey et al. 2005). Abundance of *nifH* genes found in one copepod (30 to 1.7 × 10^3^ copies ind^−1^) was lower than that found in 1 L of seawater.

Copepods are among the most abundant of invertebrates in marine mesozooplankton (Longhurst 1985), and are responsible for consuming most oceanic phytoplankton. Any gut microflora would be continuously supplied with energy‐rich substrata for microbial metabolism. Although the nitrogenase enzyme is inhibited by oxygen, the anoxic conditions found within the copepod gut could prevent enzyme deactivation, rendering the gut an ideal environment for nitrogen fixation. In general, N_2_ fixation in the ocean is limited by iron availability (Falkowski 1997). Copepod gut tracts undergo pH (Patel and Sprott 1990) and redox changes during feeding and digestion that could be important for increasing the bioavailability of trace elements (such as iron) for N_2_‐fixers. The feeding habits of copepods solubilize phytoplankton cellular iron (Hutchins et al. 1995), therefore providing a suitable mechanism for making iron available to N_2_ fixing gut microflora.

To our knowledge, this is a first systematic study of copepod or zooplankton‐associated diazotrophs, covering a large area of the Pacific Ocean. All *nifH*‐positive copepod samples with diverse N_2_ fixing prokaryotes were collected from tropical and subtropical waters throughout the Pacific Ocean (Fig. 1). Although our study found no species‐specific relationship between individual copepod and bacterial texa in our research, we do document presence, diversity and the abundance of open‐ocean diazotrophs associated with marine copepods. In order to reveal the contribution of these zooplankton‐associated diazotrophs to oceanic nitrogen cycles, the determination of N_2_ fixation rates associating with individual copepods should be required in a further study.

## Conflict of Interest

None declared.

## Supporting information


**Table S1.** Numbers of analyzed zooplankton samples. Note: only one *nifH*‐positive copepod was found during cruises MR‐11‐2 and KH‐11‐10; only copepods were analyzed from cruises KS‐13‐T2 and KH‐13‐7.
**Table S2.** Taxonomic identity of zooplankton samples from Pacific Ocean stations.*Copepod (genus).
**Figure S1.** Photomicrographs of three major *nifH*‐positive copepod genus: A, *Pontella*; B, *Euchaeta*; C, *Pleuromamma*.
