# Supplementary material for: Possible association of diazotrophs with marine zooplankton in the Pacific Ocean
Source: Microbiologyopen. 2016 Jun 28;5(6):1016–26. doi: 10.1002/mbo3.385 (PMC5221459; doi:10.1002/mbo3.385)
Supplement: Supplementary file 1 — Table S1. Numbers of analyzed zooplankton samples. Note: only one nifH‐positive copepod was found during cruises MR‐11‐2 and KH‐11‐10; only copepods were analyzed from cruises KS‐13‐T2 and KH‐13‐7. Table S2. Taxonomic identity of zooplankton samples from Pacific Ocean stations.*Copepod (genus). Figure S1. Photomicrographs of three major nifH‐positive copepod genus: A, Pontella; B, Euchaeta; C, Pleuromamma. [file MBO3-5-1016-s001.doc]

Supplementary information

Table S1. Numbers of analyzed zooplankton samples. Note: only one *nifH*-positive copepod was found during cruises MR-11-2 and KH-11-10; only copepods were analyzed from cruises KH-13-T2 and KH-13-7.

| Cruise | Station | Copepod | Amphipod | Arrow worm | Krill | Pteropoda | Doliolida | Mollusk | Shrimp | Salp | Polychaete |
| --- | --- | --- | --- | --- | --- | --- | --- | --- | --- | --- | --- |
| MR-11-2 | S1 | 5 | 2 | 1 | 1 | - | 1 | - | - | - | - |
|  | K2 | 19 | 4 | 3 | 3 | 2 | 2 | 1 | 2 | 1 | 5 |
| KH-11-10 | ALOHA | 23 | 3 | 4 | 4 | - | 1 | 2 | 3 | 1 | 4 |
| KS-13-T2 | KT | 10 | - | - | - | - | - | - | - | - | - |
| KH-13-7 | St-0 | 6 | - | - | - | - | - | - | - | - | - |
|  | St-2 | 1 | - | - | - | - | - | - | - | - | - |
|  | St-5 | 6 | - | - | - | - | - | - | - | - | - |
|  | St-6 | 4 | - | - | - | - | - | - | - | - | - |
|  | St-7 | 1 | - | - | - | - | - | - | - | - | - |
|  | St-8 | 8 | - | - | - | - | - | - | - | - | - |
|  | St-I | 3 | - | - | - | - | - | - | - | - | - |
|  | St-U | 4 | - | - | - | - | - | - | - | - | - |
|  | Total | 90 | 9 | 8 | 8 | 2 | 4 | 3 | 5 | 2 | 9 |

Table S2. Taxonomic identity of zooplankton samples from Pacific Ocean stations.*Copepod (genus).

| Station | Sample | Taxon | Station | Sample | Taxon | Station | Sample | Taxon |
| --- | --- | --- | --- | --- | --- | --- | --- | --- |
| S1 | A-1 | Amphipod |  | B-20 | Copepod | ALOHA | AL-4 | Copepod |
|  | A-2 | Copepod |  | B-21 | Mollusk |  | AL-5 | Copepod |
|  | A-3 | Doliolida |  | B-22 | Polychaeta |  | AL-6 | Copepod |
|  | A-4 | Copepod |  | B-23 | Polychaeta |  | AL-7 | Amphipod |
|  | A-5 | Krill |  | B-24 | Polychaeta |  | AL-8 | Arrow worm |
|  | A-6 | Amphipod |  | B-25 | Copepod |  | AL-9 | Arrow worm |
|  | A-7 | Copepod |  | B-26 | Copepod |  | AL-10 | Amphipod |
|  | A-8 | Copepod |  | B-27 | Amphipod |  | AL-11 | Polychaeta |
|  | A-9 | Copepod |  | B-28 | Amphipod |  | AL-12 | Copepod |
|  | A-10 | Arrow worm |  | B-29 | Copepod |  | AL-13 | Copepod |
| K2 | B-1 | Polychaeta |  | B-30 | Pteropod |  | AL-14 | Copepod |
|  | B-2 | Copepod |  | B-31 | Krill |  | AL-15 | Copepod |
|  | B-3 | Arrow worm |  | B-32 | Copepod |  | AL-16 | Copepod |
|  | B-4 | Krill |  | B-33 | Arrow worm |  | AL-17 | Copepod |
|  | B-5 | Arrow worm |  | B-33 | Copepod |  | AL-18 | Pteropoda |
|  | B-6 | Krill |  | B-34 | Doliolida |  | AL-19 | Copepod |
|  | B-7 | Copepod |  | B-35 | Doliolida |  | AL-20 | Copepod |
|  | B-8 | Copepod |  | B-36 | Shrimp larvae |  | AL-21 | Copepod |
|  | B-9 | Copepod |  | B-37 | Shrimp larvae |  | AL-21 | Copepod |
|  | B-10 | Amphipod |  | B-38 | Polychaeta |  | AL-22 | Shrimp larvae |
|  | B-11 | Amphipod |  | B-39 | Copepod |  | AL-23 | Shrimp larvae |
|  | B-12 | Copepod |  | B-40 | Copepod |  | AL-24 | Shrimp larvae |
|  | B-13 | Pteropoda |  | B-41 | Copepod |  | AL-25 | Copepod |
|  | B-14 | Copepod |  | B-42 | Copepod |  | AL-26 | Copepod |
|  | B-15 | Copepod | ALOHA | AL-1 | Krill |  | AL-27 | Amphipod |
|  | B-16 | Copepod |  | AL-2 | Krill |  | AL-28 | Copepod |
|  | B-17 | Copepod |  | AL-3 | Copepod |  | AL-29 | Amphipod |

| Station | Sample | Taxon | Station | Sample | Taxon* | Station | Sample | Taxon* |
| --- | --- | --- | --- | --- | --- | --- | --- | --- |
| ALOHA | AL-30 | Copepod | KT | KT-1 | *Labidocera* |  | KH-32 | *Pleuromamma* |
|  | AL-31 | Copepod |  | KT-2 | *Labidocera* | ST-6 | KH-15 | *Scolecithricella* |
|  | AL-32 | Copepod |  | KT-3 | *Corycaeus* |  | KH-16 | *Pleuromamma* |
|  | AL-33 | Polychaeta |  | KT-4 | *Labidocera* |  | KH-17 | *Calanus* |
|  | AL-34 | Polychaeta |  | KT-5 | *Labidocera* |  | KH-18 | *Clausocalanus* |
|  | AL-35 | Salp |  | KT-6 | *Labidocera* | ST-7 | KH-24 | *Pleuromamma* |
|  | AL-36 | Arrow worm |  | KT-7 | *Labidocera* | ST-8 | KH-5 | *Clausocalanus* |
|  | AL-37 | Polychaeta |  | KT-8 | *Labidocera* |  | KH-6 | *Pleuromamma* |
|  | AL-38 | Krill |  | KT-9 | *Labidocera* |  | KH-7 | *Acarita* |
|  | AL-39 | Krill |  | KT-33 | *Labidocera* |  | KH-8 | Aetideidae |
|  | AL-40 | Arrow worm | ST-0 | KH-19 | *Euchaeta* |  | KH-9 | *Oncaea* |
|  | AL-41 | Mollusk |  | KH-20 | *Euchaeta* |  | KH-10 | *Eucalanus* |
|  | AL-42 | Mollusk |  | KH-21 | *Euchaeta* |  | KH-11 | *Euchaeta* |
|  | AL-43 | Doliolida |  | KH-34 | *Eucheata* |  | KH-25 | *Pleuromamma* |
|  | AL-44 | Copepod |  | KH-35 | *Euchaeta* | ST-I | KH-12 | *Pontella* |
|  | AL-45 | Copepod |  | KH-37 | *Euchaeta* |  | KH-13 | *Pontella* |
|  |  |  | ST-2 | KH-22 | *Pontella* |  | KH-14 | *Calanus* |
|  |  |  | ST-5 | KH-27 | *Pleuromamma* | ST-U | KH-1 | *Pleuromamma* |
|  |  |  |  | KH-28 | *Pleuromamma* |  | KH-2 | *Pleuromamma* |
|  |  |  |  | KH-29 | *Pleuromamma* |  | KH-3 | *Pleuromamma* |
|  |  |  |  | KH-30 | *Pleuromamma* |  | KH-4 | *Pleuromamma* |
|  |  |  |  | KH-31 | *Pleuromamma* |  |  |  |

**A**


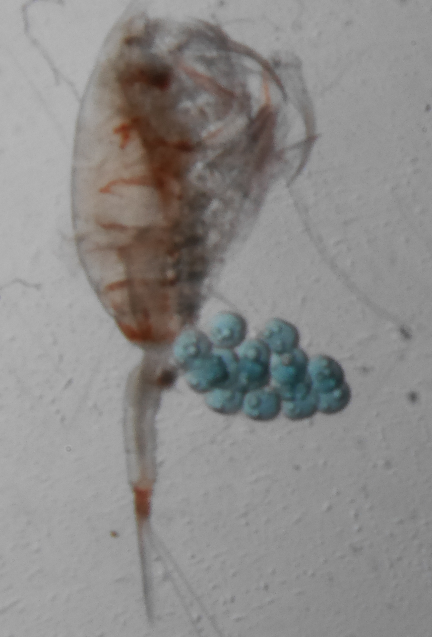

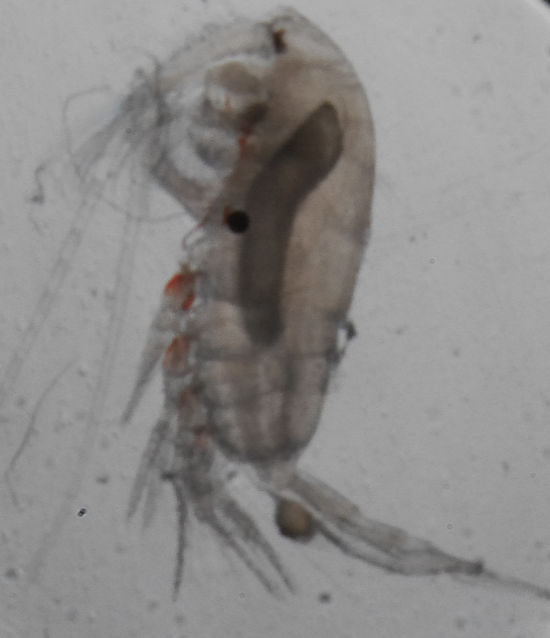


B

C

Figure S1

**Caption**. Photomicrographs of three major *nifH*-positive copepod genus: A, *Pontella*; B, *Euchaeta*; C, *Pleuromamma.*
